# Activin-A urine levels correlate with radiological patterns in preterm infants complicated by intraventricular hemorrhage

**DOI:** 10.1186/s13052-025-01938-4

**Published:** 2025-03-20

**Authors:** Giuseppe Lapergola, Giorgia Gasparroni, Alessandro Graziosi, Darek Gruzfeld, Bashir Moataza, Hanna Aboulgar, Hala Mufeed, Iman Iskander, Giovanni Livolti, Fabio Galvano, Gabriella Levantini, Ebe D’Adamo, Adele Patrizia Primavera, Elisabetta Barbante, Rita Salomone, Claudia D’Egidio, Chiara Strozzi, Antonio Maconi, Danilo A. W. Gavilanes, Ali Saber Abdelhameed, Diego Gazzolo

**Affiliations:** 1 Neonatal Intensive Care Unit, G. d’Annunzio University, Chieti, Italy; 2 Department of Neonatology and Intensive Care of Neonate, Warsaw University, Warsaw, Poland; 3 Department of Neonatology, Cairo University, Cairo, Egypt; 4 Department of Biological Chemistry, Medical Chemistry and Molecular Biology, University of Catania, Catania, Italy; 5 Ospedale Cardinal Massaia, Asti, Italy; 6 Social Security Administration Development and Promotion of Scientific Research Unit, SS Antonio, Biagio and C. Arrigo Hospital, Alessandria, Italy; 7 Department of Pediatrics and Neonatology, Maastricht University, Maastricht, The Netherlands; 8 Department of Pharmaceutical Chemistry, College of Pharmacy, King Saud University, Riyadh, Saudi Arabia

**Keywords:** Cerebral ultrasound, Intraventricular hemorrhage, Magnetic resonance imaging, Preterm infants, Activin A

## Abstract

**Background:**

To validate the role of Activin A in the early diagnosis and prognosis of preterm newborns at risk for intraventricular hemorrhage and neurological sequelae by means of cerebral ultrasound and magnetic resonance imaging (MRI), currently considered standard of care procedures.

**Methods:**

We conducted an observational case–control study in 46 preterm newborns, 23 with intraventricular hemorrhage (IVH group) and 23 controls matched for gestational age. Standard clinical, laboratory, cerebral ultrasound monitoring procedures and Activin A urine measurement were performed at four time-points (first void, 24, 48, 96 h) after birth. Cerebral MRI was performed at 40–42 weeks of corrected gestational age.

**Results:**

Elevated (*P* < 0.001, for all) Activin A levels were observed in the IVH group at all monitoring time-point. Activin A correlated (*P* < 0.05, for all) with intraventricular hemorrhage grade on cerebral ultrasound. At the cut-off of 0.08 pg/mL Activin A at 48-h achieved the best sensitivity, specificity, positive/negative predictive values as early predictor of an abnormal MRI pattern (area under the curve: 0.93).

**Conclusions:**

The present data showing a correlation among Activin A, cerebral ultrasound and MRI provide further support to Activin A inclusion in clinical daily management of cases at risk for intraventricular hemorrhage and adverse neurological outcome.

## Background

Despite technological improvements in perinatal care, preterm birth still represents the most important cause of perinatal mortality and morbidity, accounting for 5–9% in Europe and up to 12–13% in the USA [1, 2]. In this regard, intraventricular hemorrhage (IVH) remains the main complication of prematurity, leading to adverse short-long term neurological outcomes [3]. Obtaining a prompt diagnosis of IVH is still an unsolved issue: when injury is at a sub-clinical stage standard diagnostic procedures can be silent or unavailable [4]. Therefore, the assessment in biological fluids of biomarkers (BM) denoting central nervous system (CNS) development/damage could be especially useful. Recently, the Food and Drug Administration, the European Medicines Agency and the National Institutes of Health promoted research for the inclusion of BM in clinical daily practice [5]. In this regard, BM have to fulfill a series of requirements including the possibility of monitoring the progression and the extension of the disease through correlation with so-called standard-of -care procedures such as cerebral ultrasound (CUS) and magnetic resonance imaging (MRI).

Among a suite of potential BM currently under investigation, Activin A (AcA) has been indicated as a useful tool for perinatal CNS monitoring. AcA is a member of the TGF-beta superfamily involved in several cellular processes, such as neuronal differentiation and survival after hypoxia insult [6–8]. Elevated AcA levels in several biological fluids have been found in infants complicated by IVH, perinatal asphyxia and hypoxic ischemic encephalopathy [9–12]. However, data on the correlation between longitudinal AcA levels in biological fluids and CUS and MRI patterns suggesting CNS damage following IVH are still lacking.

Therefore, it was the aim of the Cooperative Multitask against Brain Injury of Neonates International Network (CoMBINe) to investigate in a cohort of preterm newborns (PN) whether longitudinal AcA urine levels: i) changed in PN complicated by IVH, and ii) correlated with CUS and MRI patterns, and iii) were predictors of abnormal MRI patterns.

## Materials and methods

### Patients

Research involving human subjects complied with all relevant national regulations, institutional policies and was conducted in accordance with the tenets of the Helsinki Declaration (as revised in 2013). The local Ethic Committees of CoMBINe approved the study protocol and informed and signed consent was obtained from all parents of patients admitted to the study. From January 2016 and December 2020 we performed at our third level centers for NICUs a case–control study in a cohort of PN (< 30 weeks), either complicated or not by IVH.

**Fig. 1:**
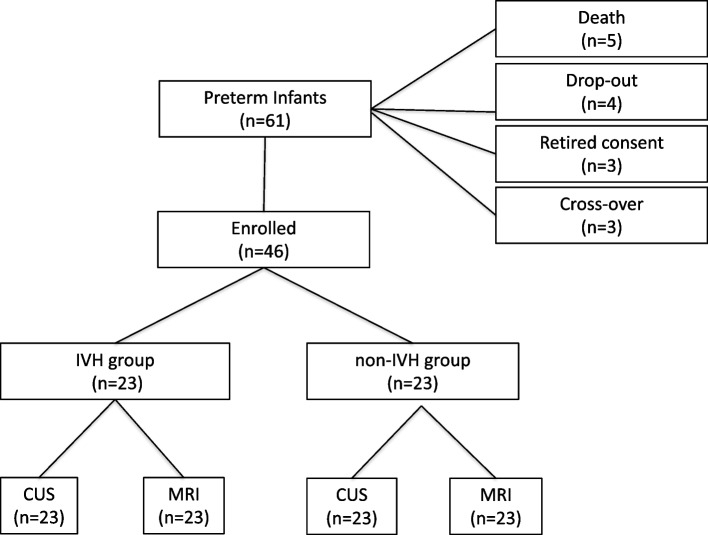
Flow chart describing the recruitment of preterm infants complicated or not by intraventricular hemorrhage (IVH), diagnosed by cerebral ultrasound scanning (CUS), and abnormal magnetic resonance imaging (MRI) patterns

Sample size determination was based on AcA urine changes occurring in IVH newborns as previously reported [13]. Assuming an increase of 0.5 SD in AcA concentrations with a α = 0.05, a two-sided test and estimating a power of 0.80 we recruited 17 PN. We added *n* = 6 cases per group to allow for mortality, dropouts, withdrawal of consent and cross-over (Fig. 1). The study population therefore consisted of a group of 23 IVH and another of 23 non-IVH infants (1 IVH vs 1 control) matched for gestational age (GA) at sampling. The sample size was calculated using nQuery Advisor® (Stonehill Corporate Center, Saugus, MA, USA) software version 5.0.

GA was determined by clinical data and by first trimester ultrasound scan. Appropriate growth was defined by the presence of ultrasonography signs (when biparietal diameter and abdominal circumference were between the 10th and the 90th percentiles), according to the nomograms of Campbell and Thoms [14], and by postnatal confirmation of a BW between the 10th and 90th percentiles, according to our population standards, corrected according to the mother’s height, weight, parity and the sex of the newborn [15].

Exclusion criteria were: CNS malformations, chromosomal abnormalities, congenital heart diseases, congenital infections and maternal drug addiction. Infants with any malformations, cardiac or hemolytic disease were also excluded from the study.

### Monitoring parameters

Standard clinical, laboratory and radiological monitoring parameters (i.e. red blood cell count; hemoglobin concentration; hematocrit rate; venous blood pH; partial pressure of oxygen and carbon dioxide, pO_2,_ pCO_2_; base excess; ion concentrations; plasma glucose, urea, creatinine and bilirubinemia) were also recorded.

In PN, clinical and laboratory parameters were recorded at five time-points: at first urination (time 0, T0), 24 (time 1, T1), 48 (time 2, T2), 72 (time 3, T3) and 96 (time 4, T4) hours after birth.

### Perinatal characteristics and neonatal outcomes

The following main perinatal and neonatal outcomes were recorded in the studied groups: maternal age, the incidences of premature rupture of membranes (PPROM), chorioamnionitis and intrauterine growth retardation (IUGR) and twin pregnancies, antenatal glucocorticoids supplementation (GC), GA, BW, delivery mode, gender, Apgar scores at 1st and 5th minutes, the occurrence of respiratory distress syndrome (RDS), of pneumothorax (PNX), and of bronchopulmonary dysplasia (BPD), the need of mechanical or non-invasive respiratory support and surfactant administration, the incidence of persistent patent ductus arteriosus (PDA) requiring pharmacological treatment, retinopathy of prematurity (ROP) greater than second degree, necrotizing enterocolitis (NEC), early/late onset sepsis (EOS, LOS). Moderate/severe BPD was defined according to Jobe and Bancalari and ROP to the ROP International Committee criteria [16, 17].

### Neurological examination

Neurological examination was performed at birth and at 24, 48 and 96-h of age. Neonatal neurological conditions were classified as described by Prechtl [18]. Each infant was assigned to one of the three diagnostic groups: normal, suspect or abnormal, in accordance with the classification used by Jurgens–van der Zee et al. [19]. An infant was considered to be abnormal when one or more of the following neurological syndromes was unequivocally present: i) increased/decreased excitability (hyperexcitability syndrome, convulsions, apathy syndrome or coma); ii) increased/decreased motility (hyperkinesia/hypokinesia); iii) increased or decreased tonus (hypertonia/hypotonia); iv) asymmetries (peripheral/central); v) defects of the CNS, and vi) any combination of the above. When indications of the presence of a syndrome were inconclusive or if only isolated symptoms were present the case was classified as suspect.

### Cranial assessment

CUS was performed during the first 72-h of life in all the study population. Recordings were performed by real-time ultrasound machine (Acuson 128SP5 Mountain View CA, USA) at the predetermined monitoring time-points. A single examiner who did not know the results of the urine test and clinical data reviewed the images. IVH was classified according to the criteria of Papile et al. [20].

### Activin A measurement

Activin A levels in urine (0.5 mL) were collected at the five predetermined monitoring time-points by using a standard urine bag collector. Samples were centrifuged at 900 g for 10 min, and supernatants stored at 70°C. AcA was measured by an ELISA assay (Bio-Rad Laboratories, Segrate, Italy), as previously described [13] by an operator blind to clinical pattern. The assay detection limit was 10 ng/L, and samples were tested in a single run with intra- and inter-assay CVs of 2.5% and 3.0%, respectively.

### Cerebral MRI

MRI was performed at 40–42 corrected GA by means of a 1.5-T scanner. Standard sequences included sagittal and axial spin-echo T1, double-acquisition axial fast-spin echo T2 proton density, coronal fast-spin echo T2, and axial diffusion-weighted images. A single operator blinded to clinical and laboratory data reviewed the images.

A standardized scoring system (K-score) was used to evaluate cerebral white matter (WM), cortical gray matter (CGM), deep gray matter (DGM), cerebellum abnormalities (CER) and global brain abnormality (GLOB) [21].

### Statistical analysis

AcA urine levels were expressed as the median and 5–95% coefficient intervals (CI). Data were analyzed for statistically significant differences between groups by a student’s t-test and a Mann–Whitney U two-sided test when not normally distributed. Comparison between proportions was performed with Fisher’s exact test. Correlations between AcA at different monitoring timepoints and K-score were calculated by linear regression analysis.

Sensitivity, specificity and positive and negative predictive values (PPV, NPV) of AcA as diagnostic test for the detection of an abnormal MRI pattern in IVH newborns were assessed using the receiver operating characteristic curve (ROC) test. The probability of developing brain damage by means of MRI when neither, one, or both tests were positive (higher than the cut-off point) was estimated and compared with the pretest probability, defined as the prevalence of brain damage in the whole group of newborns. Comparisons between ROC curves were performed by using Hanley and McNeil test. Data was analyzed using Sigma-Stat 8.0 (SPSS Inc., Chicago, IL, USA). Statistical significance was set at *P* < 0.05.

## Results

### Main outcomes measures

Table 1 shows perinatal characteristics of the studied groups. No significant differences (*P* > 0.05, for all) were observed in the two studied groups regarding maternal age, the incidences of PPROM, chorioamnionitis, IUGR, twin pregnancies as well as the rate of maternal GC antenatal supplementation. Identically, GA, BW, delivery mode and gender were superimposable (*P* > 0.05, for all) in the two groups, as well as Apgar scores at 1–5’. Among the several main outcomes there were no differences (*P* > 0.05, for all) between studied groups regarding the incidences of RDS, the need for mechanical ventilation and surfactant therapy, PNX, BPD, PDA, NEC, ROP, EOS, LOS as well as neurological examination at T0-T4 time-points and at discharge from hospital.

**Table 1:** Main perinatal characteristics, outcome measures and laboratory parameters in preterm infants complicated (IVH) or not (non-IVH) by intraventricular hemorrhage

Parameter	non-IVH (*n* = 23)	IVH (*n* = 23)
*Perinatal/Neonatal Characteristics*		
Maternal Age (yrs)	29 ± 4	28 ± 5
PPROM	12/23	13/23
Corioamnionitis	9/23	11/23
IUGR	2/23	2/23
Twins	2/23	2/23
GC	14/23	13/23
GA (wks)	29 ± 1	28 ± 2
BW (g)	1508 ± 475	1488 ± 502
Delivery mode (CS/VD)	14/9	15/23
Gender (M/F)	16/23	17/23
Apgar score at 1’ < 7	11/23	13/23
Apgar score at 5’ < 7	4/23	5/23
*Neonatal Outcomes*		
RDS	13/23	16/23
PNX	1/23	1/23
BPD	0/40	0/40
Mechanical ventilation	9/23	8/23
Surfactant therapy	20/23	21/23
PDA	2/23	4/23
NEC	0/23	1/23
ROP	1/230	2/23
EOS	4/23	4/23
LOS	5/23	6/23
*Neurological examination*		
normal/suspect/abnormal T0-T4	12/10/1	15/6/2
normal/suspect/abnormal at discharge	18/5/0	13/5/5
*Laboratory analytes*		
Red blood cell count (10^12^/L)	4.1 ± 0.4	4.0 ± 0.7
Hemoglobin (g/L)	141 ± 2.6	141 ± 2.6
Hematocrit rate (%)	41 ± 0.2	41 ± 0.2
Venous blood pH > 7.20	40/40	40/40
pCO_2_ (mmHg)	42.3 ± 3.8	42.3 ± 3.8
pO_2_ (mmHg)	43.4 ± 0.95	43.4 ± 0.95
Base excess	2.7 ± 0.8	2.7 ± 0.8
Na^+^ (mmol/L)	138.5 ± 0.5	138.5 ± 0.5
Ca^++^ (mmol/L)	1.13 ± 0.02	1.13 ± 0.02
K^+^ (mmol/L)	3.5 ± 0.15	3.5 ± 0.15
Plasma glucose (mmol/L)	4.8 ± 0.5	4.8 ± 0.5
Urea (mg/dl)	38 ± 8.1	38 ± 8.1
Creatinine (mg/dl)	0.9 ± 0.15	0.9 ± 0.15
Bilirubinemia (mg/dl)	9.1 ± 1.3	9.1 ± 1.3

*Abbreviations*: *PPROM* Premature rupture of membrane, *IUGR* Intrauterine growth retardation, *GC* Antenatal glucocorticoids, *GA* Gestational age, *BW* Birth-weight, *CS* Caesarean section, *VD* Vaginal delivery, *M* male, *F* Female, *RDS* Respiratory distress syndrome, *PNX* Pneumothorax, *BPD* Bronchopulmonary dysplasia, *PDA* Patent ductus arteriosus, *NEC* Necrotizing enterocolitis, *ROP* Retinopathy of prematurity, *EOS* Early onset sepsis, *LOS* Late onset sepsis, *pCO*_*2*_ Partial carbon dioxide pressure, *pO*_*2*_ Partial oxygen pressure

Laboratory parameters recorded at admission to the NICU such as venous blood red blood cell count, hemoglobin, hematocrit rate, pH, pCO_2_, pO_2_, base excess, ions, glucose, urea, creatinine and bilirubin levels did not differ (*P* > 0.05, for all) between groups (Table 1).

### CUS pattern

CUS was performed in the studied groups according to current guidelines. As expected no IVH was observed in the non-IVH group whilst in the IVH groups Grade I IVH occurred in 5 PN, Grade II in 13 PN, Grade III in 3 PN, and Grade IV in 2 PN, respectively.

### MRI pattern

MRI results in the two studied groups are reported in Table 2. As expected, significantly higher K-scores (*P* < 0.05, for all) were observed in the IVH than non-IVH infants in terms of WM, CGM, DGM, CER and GLOB values.

**Table 2:** Magnetic resonance imaging results in infants complicated or not by intraventricular hemorrhage (IVH). *P < 0.05 vs. control

	**non-IVH** **(*n* = 23)**			**IVH** **(*n* = 23)**		
	**Median**	**Min**	**Max**	**Median**	**Min**	**Max**
WM	0	0	4	8*	3	10
CGM	0	0	3	7*	4	9
DCM	0	0	3	3*	2	4
CER	0	0	3	4*	2	5
GLOB	0	0	10	23*	14	25

*Abbreviations*: *IVH* Intraventricular hemorrhage, *WM* White matter, *CGM* Cerebral grey matter, *DGM* Deep grey matter, *CER* Cerebellum, *GLOB* Global brain abnormality score

### Activin A measurement

AcA urine levels were measurable in all recruited infants. In the non-IVH group AcA showed a flat trend from T0 to T4 and no differences (*P* > 0.05, for all) among different monitoring time-points were observed. An identical AcA pattern was shown in the IVH group and no differences (*P* > 0.05, for all) at all monitoring time-points were detectable.

**Fig. 2:**
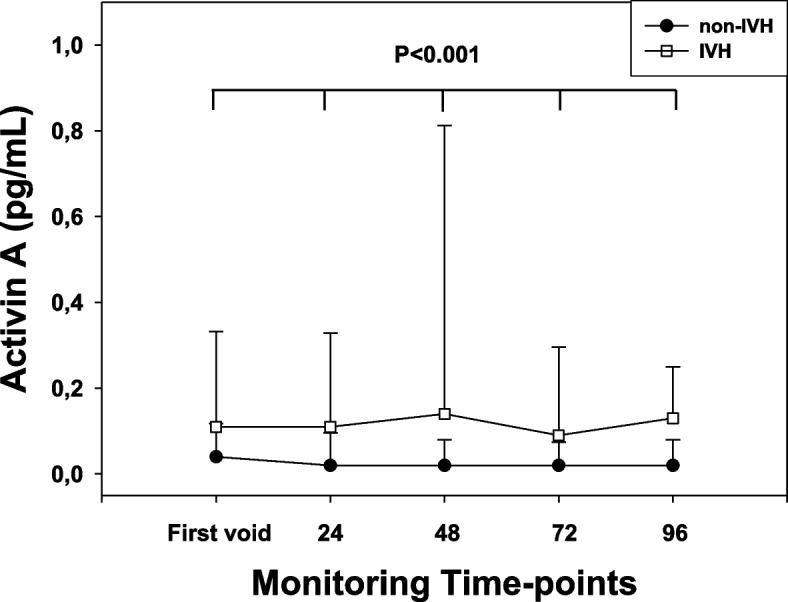
Activin A urine levels (pg/mL) recorded at the pre-determined monitoring time-points at first void (T0), 24 (T1), 48 (T2), 72 (T3) and 96 h (T4) from birth, in preterm newborns complicated or not (non-IVH) by intraventricular hemorrhage (IVH). AcA was significantly (*P* < 0.05, for all) higher in the IVH group at T0-T4. Values are expressed as median and interquartile ranges

When AcA was compared between non-IVH and IVH groups, higher AcA levels (*P* < 0.05, for all) were observed at all monitoring time-points (T0-T4) in the IVH infants (Fig. 2). Notably the highest AcA levels were observed in PN showing at CUS pattern suggestive of IVH grade III and IV (data not shown).

### Activin A and magnetic resonance imaging correlations

A positive significant correlation (*P* < 0.05, for all) between AcA at T1-T4 time-points and all MRI parameters was found. Conversely, at T0 AcA positively correlated (*P* < 0.05, for all) with CGM, CER and GLOB, whilst no significant correlations (*P* > 0.05, for both) were observed with WM and DGM.

### ROC curves analysis

**Fig. 3:**
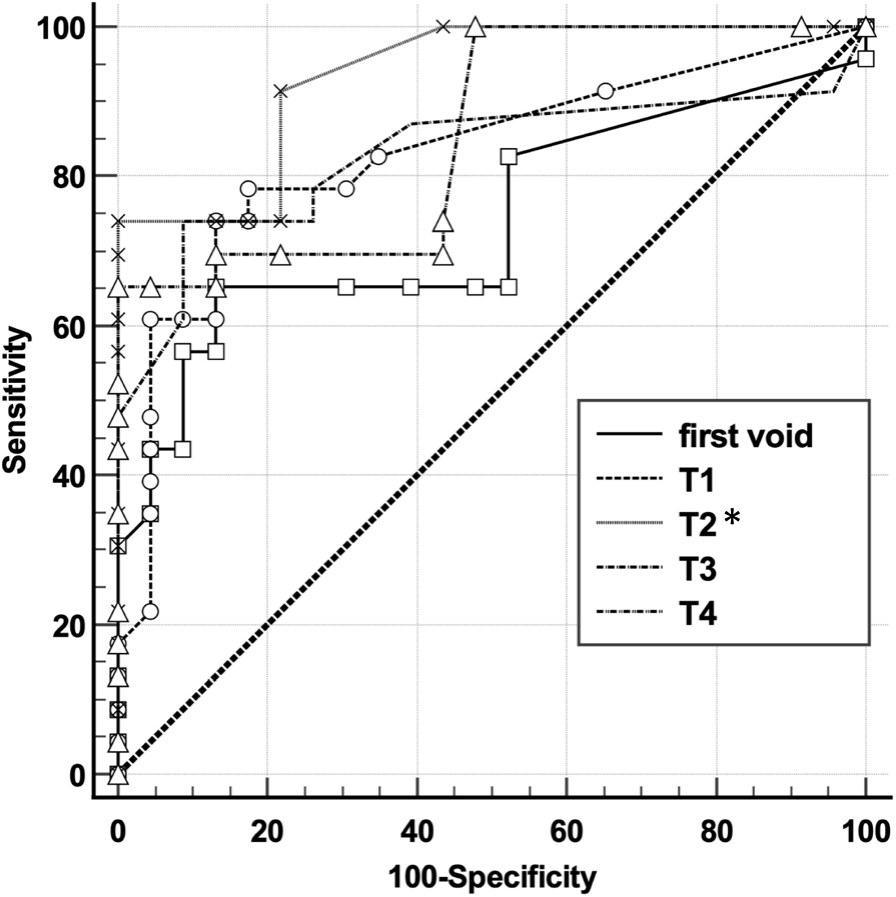
Receiver operating characteristic curve (ROC) of AcA urine levels measured at first void (T0), 24 (T1), 48 (T2), 72 (T3) and 96 h (T4) from birth, in preterm newborns complicated or not by intraventricular hemorrhage for the early prediction for abnormal magnetic resonance imaging pattern. At T2 AcA, at the cut-off of 0.08 pg/mL, achieved the best sensitivity (CI _5–95%_: 74, 52–90), specificity (CI _5–95%_: 100, 85–100), positive (CI _5–95%_: 100) and negative predictive value (CI _5–95%_: 79, 66–88) as best predictor of abnormal magnetic resonance imaging pattern (area under the ROC curve: CI _5–95%_: 0.93, 0.82–0.99). *P < 0.05 T2 vs. T0

ROC curve analysis results are reported in Table 3. At the cut-off of 0.08 pg/mL, chosen from the ROC curve, AcA at T2 (48-h), achieved the best sensitivity, specificity, PPV and NPV as best predictor of abnormal MRI pattern (AUC: 0.93). When we compared ROC curves at T0-T4, significantly higher (*P* < 0.05) values were found between T2 and T0, whilst no differences (*P* > 0.05, for all) were observed among T0, T1, T3 and T4, respectively (Fig. 3).

**Table 3:** Prediction of pathological cerebral resonance imaging in preterm infants complicated or not by intraventricular hemorrhage based on urine Activin A concentrations (pg/mL)

Time-point	cut-off (pg/mL)	Sensitivity (CI_5–95%_)	Specificity (CI_5–95%_)	PPV (CI_5–95%_)	NPV (CI_5–95%_)	AUC (CI_5–95%_)
First void, T0	> 0.08	65(43–87)	87(66–97)	83(63–94)	71(58–82)	0.74(0.59–0.86)
24 h, T1	> 0.04	78(56–93)	83(61–95)	82(64–92)	79(63–89)	0.83(0.69–0.92)
48 h, T2	> 0.08	74(52–90)	100(85–100)	100	79(66–88)	0.93(0.82–0.99)
72 h, T3	> 0.05	74(52–90)	91(72–99)	90(69–97)	78(64–88)	0.83(0.69–0.92)
96 h, T4	> 0.09	65(43–84)	100(85–100)	100	74(62–83)	0.86(0.72-.094)

*Abbreviations*: *PPV* Positive predictive value, *NPV* Negative predictive value, *CI* Coefficient interval

## Discussion

The worldwide incidence of IVH ranges from 3 to 45% of premature births, with severe grades (IVH III-IV) occurring in about 8% [22]. At this stage, among standard procedures no clinical, laboratory and radiological parameters constitute a reliable early diagnostic tool for IVH. Further problems regard the timing of the insult, in most cases of pre/perinatal origin, and the occurrence of cerebral bleeding within the first 3 days of life in most cases, about 50% in the first 5 h and in about 70% in the first 24 h of life [22, 23]. All in all, the possibility that a qualitative/quantitative brain constituent could be included in clinical daily practice once the criteria of official institutions have been met, is no longer remote.

In the present study we found in IVH PN higher urinary levels of a well-established brain development/damage marker, namely AcA. Furthermore, higher AcA levels correlated with abnormal CUS and MRI patterns. To the best of our knowledge this is the first report on the correlation between both AcA and CUS and AcA and MRI patterns, respectively.

The finding of AcA in high-risk infants partially matches previous observations and lends further support to the role of AcA as an early reliable marker of active CNS cell damage, measurable in unconventional biological fluids, when CUS and MRI are silent or not performed [5, 9, 11, 13, 24]. Discrepancies regard different monitoring time-points, endpoints and studied population (IVH vs. perinatal asphyxia) [11, 13, 25, 26].

The pattern of early increased AcA levels in PN later developing into IVH warrants further consideration. In particular, AcA: i) at its highest levels was observed in PN Grade III-IV IVH and correlated with MRI K-score values suggestive of a greater extension of CNS damage. The issue is noteworthy bearing in mind that about one-third of grade III-IV IVH lead to post-hemorrhagic hydrocephalus, increasing mortality and cerebral palsy rates [22], and ii) already increased in the IVH group at a stage (first void, < 2h) when both standard monitoring procedures and therapeutic strategies were silent or not been performed.

This fact should not be overlooked, considering AcA different functions after a hypoxic insult. On the one hand, data in animal models and in humans has shown that an early increase in protein levels can be an expression of an AcA-mediated protective action in an attempt to preserve neurons from a cascade of events leading to cell damage by means of necrosis and apoptosis mechanisms [8, 27–31]. On the other hand, the persistently high AcA levels, despite the protein’s short half-life (about 2 h) pointed toward ongoing CNS damage rather than a neuroprotective action [10–13, 32, 33]. This latter issue finds support in previous observations in PN and perinatal asphyxia infants [11, 13]. All in all, it is reasonable to argue for a dual role for AcA in PN complicated by IVH: neuroprotection and as a damage marker. Further multicenter studies over a wider population providing AcA longitudinal measurement at shorter intervals (i.e. 4 h) are therefore justified.

In the present study we also found that AcA urine levels correlated with MRI, to date the gold standard diagnostic test. The finding constitutes one of the main stages along the way to the inclusion of a BM in clinical daily practice. Among a suite of BM, today still under investigation, AcA can be added to a calcium binding protein, namely S100B, as a predictor of an adverse brain MRI pattern [34]. Furthermore, we found that in PN longitudinal AcA urine assessment, from first void up to 96 h from birth, was predictive of a pathological cerebral MRI pattern limited to the CGM, CER and GLOB parameters. It is noteworthy, in this respect, that for AcA at first void (< 2 h from birth) above the thresholds defined by the ROC curve analysis (> 0.08 ng/mL), the probability (PPV) of abnormal MRI pattern was as high as 83%, while it was 71% if these levels were below the threshold, with PPV and NPV that differed from the overall prevalence of an abnormal MRI pattern (50%) in the study population. Indeed, when we compared ROC curve results at different monitoring time-points AcA achieved the best performance as a predictor of abnormal cerebral MRI pattern at 48 h from birth. These findings promote the possibility of identifying newborns at higher risk of a poor MRI pattern in the first hours after birth. This issue should not be overlooked, bearing in mind that MRI, today the gold standard diagnostic test, can be performed at 40–42 GA, thus limiting its usefulness in the early detection of cases at risk [21, 22, 35]. Additional MRI limitations lie in the impossibility of transporting a critically ill neonate for an MRI test. Conversely, by measuring AcA, the identification of PN at risk of long-term brain damage sequelae can be obtained at the earliest stage, thus paving the way for better neuroprotective strategy management. Altogether, it is possible to argue that AcA can be considered suitable for inclusion in daily clinical practice since it achieved one of the main items requested by FDA, EMA and NIH. Further investigations aimed at fulfilling the remaining items established by official institutions are therefore awaited [5].

In the present study we identified a series of limitations. These mainly regarded: i) the quality of MRI recordings performed with a 1.5-T scanner instead of 3.0-T one, and ii) the need for a wider studied population. In this regard, further multicenter studies over a wider population using an up-to-date MRI device are required.

## Conclusions

In conclusion, the present data showed that the possibility to identify PN at risk of IVH and an abnormal MRI pattern is getting closer. The data paves the way for further studies aimed at promoting the assessment in non-invasive biological fluids of a suite of BM for the early diagnosis and management of high risk newborns.

## Data Availability

The datasets used and analysed during the current study are available from the corresponding author on reasonable request.

## Abbreviations

IVH: Intraventricular hemorrhage
BM: Biomarkers
CNS: Central Nervous System
CUS: Cerebral Ultrasound
MRI: Magnetic Resonance Imaging
AcA: Activin A
CoMBINe: Cooperative Multitask against Brain Injury of Neonates
PN: Preterm Newborns
GA: Gestational age
BW: Birthweight
pO_2_: Partial Oxygen Pressure
pCO_2_: Partial Carbon Dioxide Pressure
PPROM: Premature Rupture of Membranes
IUGR: Intrauterine Growth Retardation
GC: Antenatal Glucocorticoids
RDS: Respiratory Distress Syndrome
PNX: Pneumothorax
BPD: Bronchopulmonary Dysplasia
PDA: Patent Ductus Arteriosus
ROP: Retinopathy of Prematurity
NEC: Necrotizing Enterocolitis
EOS: Early Onset Sepsis
LOS: Late Onset Sepsis
WM: Cerebral White Matter
CGM: Cortical Gray Matter
DGM: Deep Gray Matter
CER: Cerebellum
GLOB: Global Brain Abnormality
CI: Coefficient Intervals
PPV: Positive Predictive Value
NPV: Negative Predictive Value
ROC: Receiver Operating Characteristic Curve

## References

[1] M.o. Dimes, PMNCH, S.t. Children, WHO, in: Born too soon: the global action report on preterm birth. Howson C, Kinney M, Lawn J, Eds. (World Health Organization, Geneva, Switzerland, 2012.

[2] Chawanpaiboon S, Vogel JP, Moller AB, Lumbiganon P, Petzold M, Hogan D, et al. Global, regional, and national estimates of levels of preterm birth in 2014: a systematic review and modelling analysis. Lancet Glob Health. 2019;7:e37–46. 30389451 10.1016/S2214-109X(18)30451-0 PMC6293055

[3] Gilard V, Tebani A, Bekri S, Marret S. Intraventricular hemorrhage in very preterm infants: a comprehensive review. J Clin Med. 2020;9:2447–59. 32751801 10.3390/jcm9082447 PMC7465819

[4] Leijser LM, de Vries LS. Preterm brain injury: germinal matrix–intraventricular hemorrhage and post-hemorrhagic ventricular dilatation. Hand Clin Neurol. 2019;162:173–99. 10.1016/B978-0-444-64029-1.00008-4 31324310

[5] Serpero LD, Bellissima V, Colivicchi M, Sabatini M, Frigiola A, Ricotti A, et al. Next generation biomarkers for brain injury. J Matern Fetal Neonatal Med. 2013;26:44–9. 24059552 10.3109/14767058.2013.829688

[6] Luisi S, Florio P, Reis FM, Petraglia F. Expression and secretion of activin A: possible physiological and clinical implications. Eur J Endocrinol. 2001;145:225–36. 11517001 10.1530/eje.0.1450225

[7] Luisi S, Calonaci G, Florio P, Lombardi I, De Felice C, Bagnoli F, et al. Identification of activin A and follistatin in human milk. Growth Factors. 2002;20:147–50. 12519018 10.1080/0897719021000042334

[8] Schubert D, Kimura H, LaCorbiere M, Vaughan J, Karr D, Fischer WH. Activin is a nerve cell survival molecule. Nature. 1990;344:868–70. 2330043 10.1038/344868a0

[9] Bersani I, Pluchinotta F, Dotta A, Savarese I, Campi F, Auriti C, et al. Early predictors of perinatal brain damage: the role of neurobiomarkers. Clin Chem Lab Med. 2020;58:471–86. 31851609 10.1515/cclm-2019-0725

[10] Florio P, Perrone S, Luisi S, Vezzosi P, Longini M, Marzocchi B, et al. Increased plasma concentrations of Activin A predict intraventricular hemorrhage in preterm newborns. Clin Chem. 2006;52:1516–21. 16740650 10.1373/clinchem.2005.065979

[11] Florio P, Luisi S, Bashir M, Torricelli M, Iskander I, Mufeed H, et al. High urinary concentrations of activin a in asphyxiated full-term newborns with moderate or severe hypoxic ischemic encephalopathy. Clin Chem. 2007;53:520–2. 17259240 10.1373/clinchem.2005.062604

[12] Florio P, Perrone S, Luisi S, Longini M, Tanganelli D, Petraglia F, et al. Activin A plasma levels at birth: an index of fetal hypoxia in preterm newborn. Pediatr Res. 2003;54:696–700. 12904593 10.1203/01.PDR.0000086905.71963.1D

[13] Sannia A, Zimmermann LJ, Gavilanes AW, Vles HJ, Calevo MG, Florio P, et al. Elevated Activin A urine levels are predictors of intraventricular haemorrhage in preterm newborns. Acta Paediatr. 2013;102:e449–54. 23808611 10.1111/apa.12332

[14] Campbell S, Thoms A. Ultrasound measurement of the fetal head to abdomen circumference ratio in the assessment of growth retardation. BJOG. 1977;84:165–74. 10.1111/j.1471-0528.1977.tb12550.x 843490

[15] Bertino E, Spada E, Occhi L, Coscia A, Giuliani F, Gagliardi L, et al. Neonatal anthropometric charts: the Italian neonatal study compared with other European studies. J Pediatr Gastroenterol Nutr. 2010;51:353–61. 20601901 10.1097/MPG.0b013e3181da213e

[16] Jobe AH, Bancalari E. Bronchopulmonary dysplasia. Am J Respir Crit Care Med. 2001;163:1723–9. 11401896 10.1164/ajrccm.163.7.2011060

[17] International Committee for the Classification of Retinopathy of Prematurity. The International classification of retinopathy of prematurity revisited. Arch Ophthalmol. 2005;123:991–9. 16009843 10.1001/archopht.123.7.991

[18] Prechtl HFR. Assessment methods for the newborn infant: a critical evaluation. In: Stratton D, editor. Psychobiology of human newborn. Chichester: Wiley; 1982. p. 21–52.

[19] Jurgens-van der Zee AD, Bierman-van Eendenburg ME, Fidler VJ, Olinga AA, Visch JH, Touwen BC, et al. Preterm birth, growth retardation and acidemia in relation to neurological abnormality of the newborn. Early Hum Dev. 1979;3:141–54. 535545 10.1016/0378-3782(79)90003-3

[20] Papile LA, Burstein J, Burstein R, Koffler H. Incidence and evolution of subependymal and intraventricular hemorrhage: a study of infants with birth weight less than 1500 gm. J Pediatr. 1978;92:529–34. 305471 10.1016/s0022-3476(78)80282-0

[21] Kidokoro H, Neil JJ, Inder TE. New MR imaging assessment tool to define brain abnormalities in very preterm infants at term. AJNR Am J Neuroradiol. 2013;34:2208–14. 23620070 10.3174/ajnr.A3521 PMC4163698

[22] Hand IL, Shellhaas RA, Milla SS. Committee on fetus and newborn, section on neurology, section on radiology. Routine Neuroimaging of the Preterm Brain. Pediatrics. 2020;146(5):e2020029082. 33106343 10.1542/peds.2020-029082

[23] Himmelmann K, Hagberg G, Beckung E, Hagberg B, Uvebrant P. The changing panorama of cerebral palsy in Sweden. IX. Prevalence and origin in the birth-year period 1995–1998. Acta Paediatr. 2005;94:287–94. 16028646 10.1111/j.1651-2227.2005.tb03071.x

[24] Brackmann FA, Alzheimer C, Trollmann R. Activin A in perinatal brain injury. Neuropediatrics. 2015;46:82–7. 25769120 10.1055/s-0035-1547345

[25] Lu H, Huang W, Chen X, Wang Q, Zhang Q, Chang M. Relationship between premature brain injury and multiple biomarkers in cord blood and amniotic fluid. J Matern-Fetal Neonatal Med. 2018;31:2898–904. 28738706 10.1080/14767058.2017.1359532

[26] Florio P, Luisi S, Bruschettini M, Grutzfeld D, Dobrzanska A, Bruschettini P, et al. Cerebrospinal fluid activin a measurement in asphyxiated full-term newborns predicts hypoxic ischemic encephalopathy. Clin Chem. 2004;50:2386–9. 15563489 10.1373/clinchem.2004.035774

[27] Wankell M, Werner S, Alzheimer C, Werner S. The roles of Activin in cyto-protection and tissue repair. Ann NY Acad Sci. 2003;995:48–58. 12814938 10.1111/j.1749-6632.2003.tb03209.x

[28] Tretter P, Hertel M, Munz B, ten Bruggencate G, Werner S, Alzheimer C. Induction of Activin A is essential for the neuroprotective action of basic fibroblast growth factor in vivo. Nat Med. 2000;6:812–5. 10888932 10.1038/77548

[29] Iwahori Y, Saito H, Torii K, Nishiyama N. Activin exerts a neurotrophic effect on cultured hippocampal neurons. Brain Res. 1997;760:52–8. 9237517 10.1016/s0006-8993(97)00275-8

[30] Krieglstein K, Suter-Crazzolara C, Fischer WH, Unsicker K. TGF-b superfamily members promote survival of midbrain dopaminergic neurons and protect them against MPP+toxicity. EMBO J. 1995;14:736–42. 7882977 10.1002/j.1460-2075.1995.tb07052.x PMC398139

[31] Florio P, Gazzolo D, Luisi S, Petraglia F. Activin A in brain injury. Adv Clin Chem. 2007;43:117–30. 17249382 10.1016/s0065-2423(06)43004-3

[32] Florio P, Frigiola A, Battista R, Abdalla Ael H, Gazzolo D, Galleri L, et al. Activin A in asphyxiated full-term newborns with hypoxic ischemic encephalopathy. Front Biosci (Elite Ed). 2010;2:36–42. 20036850 10.2741/e62

[33] Florio P, Abella RF, de la Torre T, Giamberti A, Luisi S, Butera G, et al. Perioperative activin A concentrations as a predictive marker of neurologic abnormalities in children after open heart surgery. Clin Chem. 2007;53:982–5. 17363421 10.1373/clinchem.2006.077149

[34] Gasparroni G, Graziosi A, Bersani I, Caulo M, Moataza B, Aboulgar H, et al. S100B protein, cerebral ultrasound and magnetic resonance imaging patterns in brain injured preterm infants. Clin Chem Lab Med. 2021;59:1527–34. 34008376 10.1515/cclm-2021-0278

[35] Volpe JJ. Intracranial hemorrhage: germinal matrix-intraventricular hemorrhage of premature infant. In: Neurology of the newborn, 5th ed Philadelphia: Saunders. 2018;11:403–63.

